# Comparison of liver scintigraphy and the liver-spleen contrast in Gd-EOB-DTPA-enhanced MRI on liver function tests

**DOI:** 10.1038/s41598-021-01815-0

**Published:** 2021-11-18

**Authors:** Hiroshige Mori, Hanaka Machimura, Amika Iwaya, Masaru Baba, Ken Furuya

**Affiliations:** 1 Department of Radiology, Japan Community Healthcare Organization Hokkaido Hospital, 1-8-3-18 Nakanoshima, Toyohira, Sapporo, Hokkaido 062-8618 Japan; 2 Center for Gastroenterology and Hepatology, Japan Community Healthcare Organization Hokkaido Hospital, 1-8-3-18 Nakanoshima, Toyohira, Sapporo, Hokkaido 062-8618 Japan

**Keywords:** Magnetic resonance imaging, Liver

## Abstract

The liver-spleen contrast (LSC) using hepatobiliary-phase images could replace the receptor index (LHL15) in liver scintigraphy; however, few comparative studies exist. This study aimed to verify the convertibility from LSC into LHL15. In 136 patients, the LSC, not at 20 min, but at 60 min after injecting gadolinium-ethoxybenzyl-diethylenetriaminepentaacetic acid was compared with the LHL15, albumin–bilirubin (ALBI) score, and the related laboratory parameters. The LHL15 was also compared with their biochemical tests. The correlation coefficients of LSC with LHL15, ALBI score, total bilirubin, and albumin were 0.740, –0.624, –0.606, and 0.523 (*P* < 0.00001), respectively. The correlation coefficients of LHL15 with ALBI score, total bilirubin, and albumin were –0.647, –0.553, and 0.569 (*P* < 0.00001), respectively. The linear regression equation on the estimated LHL15 (eLHL15) from LSC was eLHL15 = 0.460 · LSC + 0.727 (*P* < 0.00001) and the coefficient of determination was 0.548. Regarding a contingency table using imaging-based clinical stage classification, the degree of agreement between eLHL15 and LHL15 was 65.4%, and Cramer's V was 0.568 (*P* < 0.00001). Therefore, although the LSC may be influenced by high total bilirubin, the eLHL15 can replace the LSC as an index to evaluate liver function.

## Introduction

**Figure 1:**
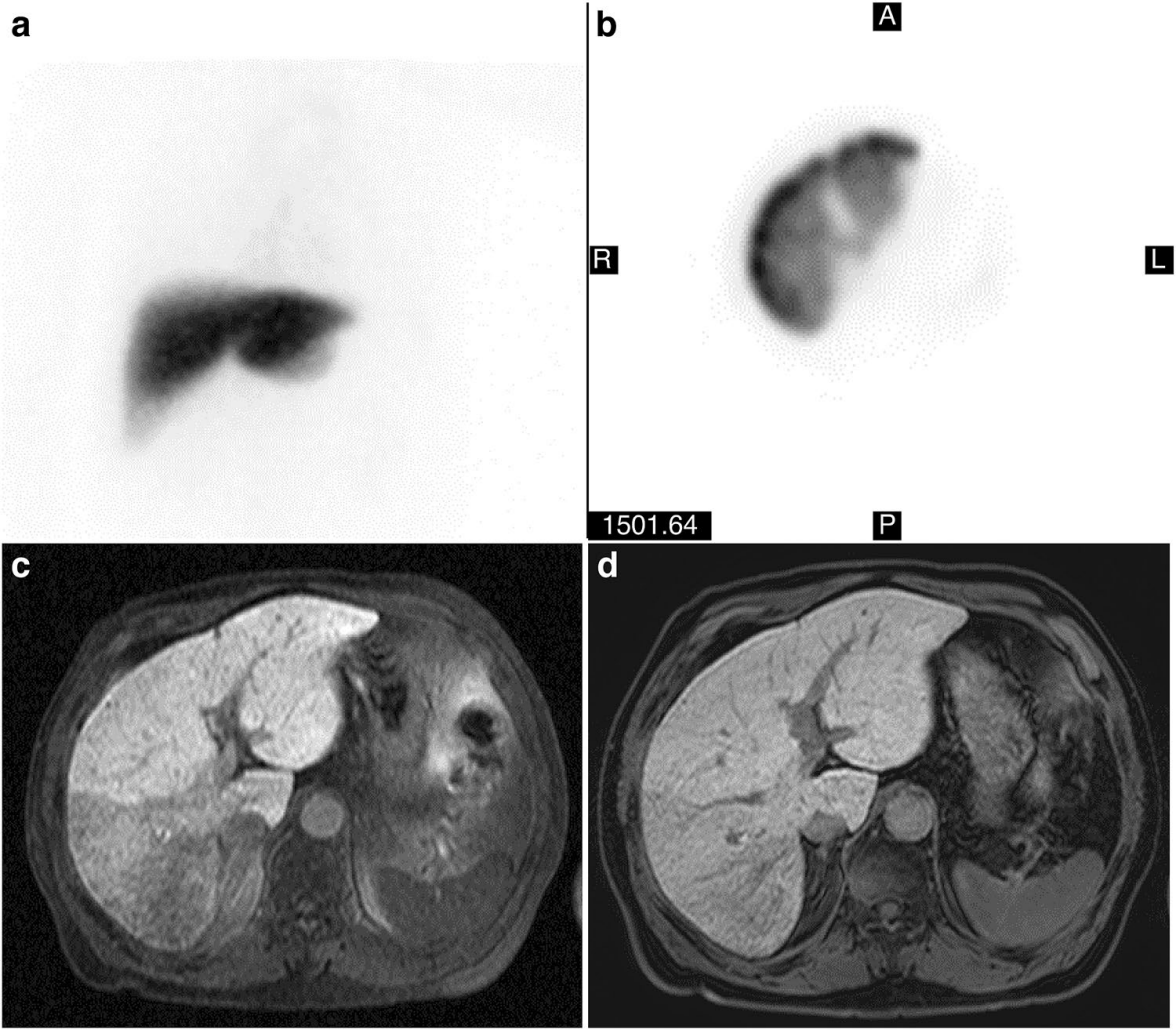
Comparison of ^99m^Tc-GSA liver scintigraphy and Gd-EOB-DTPA-enhanced MRI. (**a**) Planar image of ^99m^Tc-GSA liver scintigraphy. (**b**) Transverse image of ^99m^Tc-GSA liver scintigraphy. It is impossible to distinguish hepatic segments from this image because intrahepatic portal vein and hepatic vein were not imaged. (**c**) Gd-EOB-DTPA-enhanced MRI with a built-in body coil. A decrease in signal intensities in right posterior segments was clearly imaged, which is not found in **(a,b)** images. (**d**) Gd-EOB-DTPA-enhanced MRI with a 32ch phased array coil. The contrasts between right anterior and posterior segments decreased compared with the **(c)** image.

In invasive treatments such as hepatectomy, it is important to understand the preoperative hepatic functional reserve to avoid postoperative liver failure^1–3^. Liver function can be quantitatively evaluated by measuring the retention rate of indocyanine green (ICG) 15 min after injection^1,2^. However, in cases of non-uniform liver function, such as after portal vein embolization, the ICG test is inaccurate for estimating the function of the entire liver^2^. In addition, technical errors can arise in factors such as the ICG infusion rate or the timing of the blood draw^4^. Liver scintigraphy using ^99m^Tc-diethylenetriaminepentaacetic acid (DTPA)-galactosyl human serum albumin (GSA) is another method for evaluating liver function^2,3,5^, and aids in determining the range of partial hepatectomy using volume data^2^, as it can estimate the liver function of hepatic segments^3^. However, easy access no longer exists for ^99m^Tc formulations^6^ because the suspension producing ^99^Mo (the parent nuclide of ^99m^Tc) has become difficult to acquire from ageing nuclear reactors^6,7^. Therefore, imaging-based liver function tests using magnetic resonance imaging (MRI), which does not require radioactive tracers, have been studied as an alternative to ^99m^Tc-GSA liver scintigraphy^1,2,8^. Additionally, MRI has advantages over scintigraphy in terms of temporospatial resolution^1,2,8^ (Fig. 1a–c).

Contrast-enhanced MRI with gadolinium-ethoxybenzyl-DTPA (Gd-EOB-DTPA), which is the hepatic-specific contrast medium for MRI, is useful for evaluating liver function as well as for detecting and differentiating neoplastic hepatic lesions^1,2^. Recent trials have evaluated lobe or subsegment function using Gd-EOB-DTPA, and have investigated the preoperative prediction of the function of the future remnant liver after hepatic resection^1,9^. In liver function tests using Gd-EOB-DTPA-enhanced MRI, the liver–spleen contrast (LSC)^10,11^ in the hepatobiliary phase has been reported to be moderately or strongly correlated with the blood clearance index (HH15) and receptor index (LHL15) calculated from ^99m^Tc-GSA liver scintigraphy^11–13^. There has also been an attempt to propose the index according to the LHL15^11^. Clinicians can easily appraise a patient’s liver function by converting the LSC into the LHL15, because the evaluation standards of the HH15 and LHL15 have been supported for many years^14^. However, most studies that examined the correlation between the LSC and LHL15 did not have sufficiently large sample sizes^11–13^. In addition, the results of these correlation analyses remain controversial from the standpoint of the differences in uptake and excretion between Gd-EOB-DTPA and ^99m^Tc-GSA^12,13^.

Moreover, there is a controversy regarding contrast enhancement in the liver after the administration of Gd-EOB-DTPA. It was initially reported that the signal intensity of the liver (SI_L_) reached a plateau at 20–90 min after injection^15^. However, recent reports have stated that the SI_L_ continues to increase until 30 min after injection^8,16,17^. Furthermore, the LSC continues to increase until approximately 60 min after injection^17^, as the signal intensity of the spleen (SI_S_) continues to decrease until 30–40 min after injection^8,15,16^, in addition to the increase in SI_L_ mentioned above. Therefore, the proper timing of the hepatobiliary phase must be 60 min after injection^17^.

In the present study, the correlations between the LSC in the hepatobiliary phase at 60 min and the HH15 and LHL15 were re-evaluated using a greater number of samples compared with previous studies^11–13^; Additionally, their indices were compared with the albumin–bilirubin (ALBI) score^18^ and its related laboratory parameters. The estimated value of the LHL15 (eLHL15) was then computed using linear regression analysis formed from the LSC, and its accuracy was verified using the coefficient of determination (R^2^) and by the comparison between the eLHL15 and LHL15 based on the criteria of LHL15 for determining the severity of chronic liver disease^5^. Finally, these results were reviewed in terms of the pharmacokinetic systems of the contrast media visualizing liver function, and the feasibility of liver function tests with the eLHL15 was discussed with its clinical application.

## Methods

### Patients

**Figure 2:**
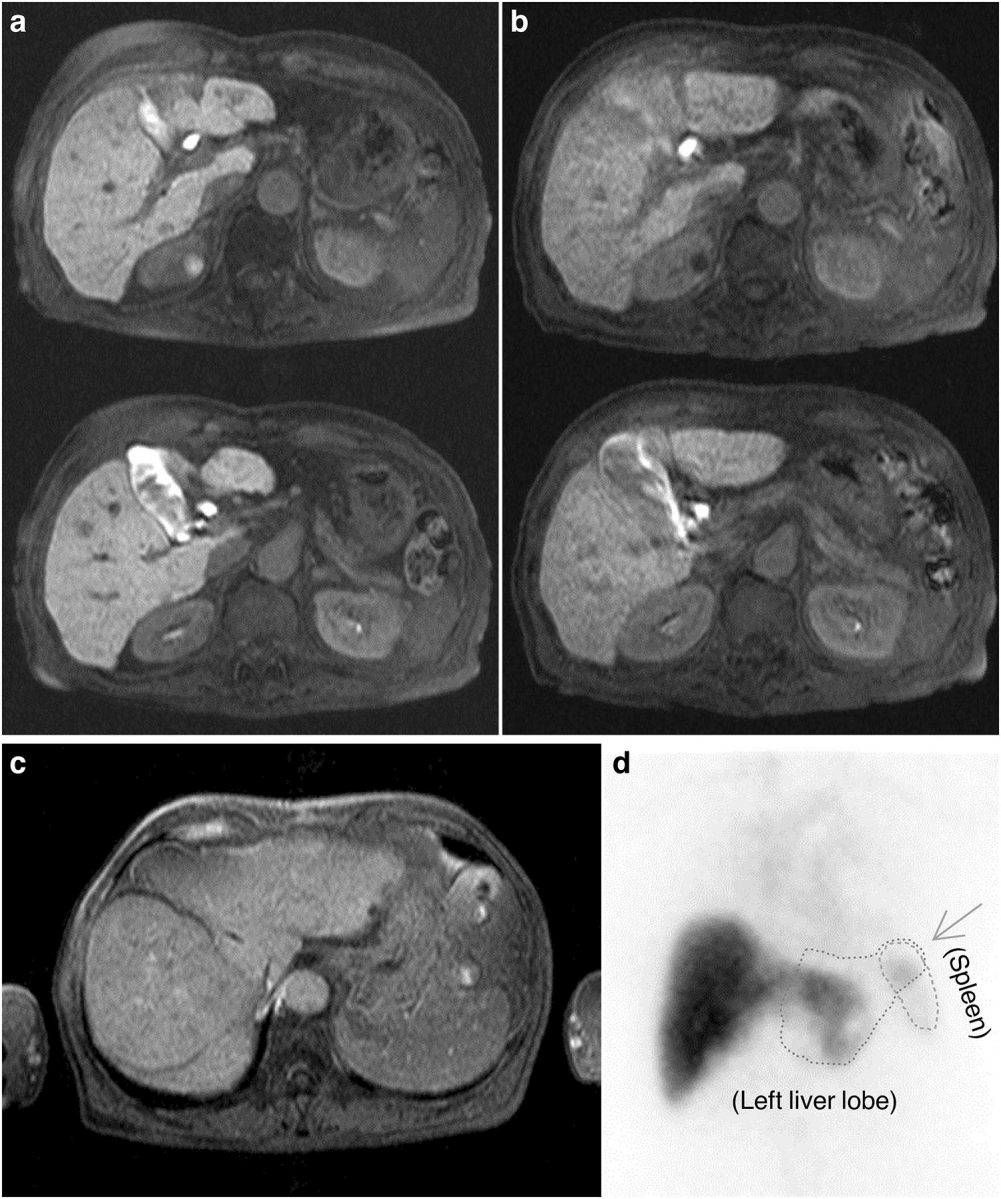
Example of image deterioration. (**a**) Images without motion artifacts (LSC = 0.294). (**b**) Images with motion artifacts (LSC = 0.125). By poor breath holding, the LSC decreases, and the white streak from a gall bladder arises for the respiratory motion artifacts. (**c**) Image of inhomogeneous fat supression. The fat supression do not work around a right anterior segment. (**d**) Image overlapping with the blood pool. The left lobe of the liver overlaps with a part of the spleen (leftward arrow).

After the exclusion of five cases where the image was deteriorated (Fig. 2), the participants comprised 136 patients (93 men and 43 women) who underwent Gd-EOB-DTPA-enhanced MRI and ^99m^Tc-GSA liver scintigraphy at our hospital within 2 weeks between 15 October 2012 and 3 February 2021. The patient age was 69.3 ± 9.6 years (mean ± standard deviation) (range: 46–93 years). Patient backgrounds are shown in Table 1. All patient data were collected retrospectively and anonymized prior to analysis. In accordance with the provision of the “Ethical guidelines for medical and health researches involving human subjects” (Ministry of Education, Culture, Sports, Science; and Technology and Ministry of Health, Labour and Welfare, Japan, Bulletin No. 3, 2014), information was posted on a notice board in our hospital requesting consent for the secondary use of medical care information. Patient information and consent were requested by disclosing the research contents to the public on the home page of the website of Japan Community Healthcare Organization Hokkaido Hospital. Informed consent was obtained from all individual participants included in the study. This study design was approved by the appropriate ethics review boards of Japan Community Healthcare Organization Hokkaido Hospital (Research Reference No. 2013-32 and No. 2018-21).

**Table 1:** Patient demographics.

Characterisitic	n
**Infection**	
Hepatitis virus (B/C)	32/24
**Liver diseases**	
Non-viral liver cirrhosis	9
Viral diseases (chronic hepatitis/liver cirrhosis)	21/18
ALD (ASH/liver cirrhosis)	12/17
NAFLD (NASH/liver cirrhosis)	9/12
**Mass**	
Carcinoma (HCC/CC)	94/11
Metastasis	33
Other mass	2
(No mass)	2
**Liver function levels**	
ALBI Grade (Grade 1/Grade 2/Grade 3)	77/55/4
**Standard hepatic function tests**	
Albumin (g/dL)	3.87 ± 0.54
Total bilirubin (mg/dL)	0.85 ± 0.51
Direct bilirubin (mg/dL)	0.30 ± 0.31

*n* number of patients, *ALD* alcoholic liver disease, *ASH* alcoholic steatohepatitis, *NAFLD* nonalcoholic fatty liver disease, *NASH* nonalcoholic steatohepatitis, *HCC* hepatocellular carcinoma, *CC* cholangiocellular carcinoma, *ALBI* albumin-bilirubin. All data of laboratory parameters are expressed as the mean ± standard deviation.

### Contrast agents and radiopharmaceuticals

For Gd-EOB-DTPA-enhanced MRI, 0.025 mmol/kg of body weight of Gd-EOB-DTPA (Primovist^®^ 0.25 mmol/mL; Bayer Yakuhin, Osaka, Japan) was injected intravenously. For ^99m^Tc-GSA liver scintigraphy, 1.0 mL of ^99m^Tc-GSA (Asialosynchis^®^ 185 MBq; Nihon Medi-Physics, Tokyo, Japan) was injected intravenously regardless of body weight.

### Imaging data acquisition

The device used for MRI was Achieva 1.5 T A-series R.2.6 (Philips Medical Systems Japan, Tokyo, Japan) with a quadrature body coil, which has the best uniformity of images in coils within the field of view (FOV) (Fig. 1c). Phased array coils were not used because the image non-uniformity correction, which is indispensable for using these coils, causes image unevenness and a decrease in contrasts (Fig. 1d)^19^. The breath-hold T_1_-weighted two-dimensional images were acquired using fast field echo [FFE] of multi slice at 20.7 s. In addition, the fat suppression on the images was carried out using the principle of selective excitation technique [ProSet], and its pulse type was 121. The pulse sequence parameters were as follows: echo time was 5.1 ms, repetition time was 126 ms, flip angle was 80°, number of excitations was 1, and band width was 313.8 Hz/pixel. The imaging parameters were as follows: FOV was 420 mm, matrix was 256 × 160 (frequency × phase), k-space trajectory was linear, scan percentage was 62.5%, and phase FOV was 100%. The other parameters on slices were as follows: slice thickness was 6.5 mm, slice gap was 3.5 mm, slice scan order was interleaved, and slice number was 7. Hepatobiliary-phase images were acquired, not at 20 min, but at 60 min after intravenous injection^17^.

The device used for scintigraphy was INFINIA Functional Imaging Scanner (General Electric Healthcare, Tokyo, Japan) with a low-energy high-resolution collimator. The scan parameters were as follows: frame rate was 30 s/frame, frame number was 40 frame, matrix was 128 × 128, energy level was 140 keV, window width was ± 10%, and continuous scan time was 20 min.

### Image analysis

The measurement of LSC was performed using Basic Viewing (Philips Medical Systems Japan, Tokyo, Japan). The region of interest (ROI) was set with a rectangle of approximately 50 pixels in order to suppress statistical fluctuations^11^. To minimize the signal change inside the ROIs, the ROIs of the SI_L_ and SI_S_ were placed on the areas with the smallest standard deviation, avoiding large vessels, masses, and artefacts^11^. The LSC was calculated using the SI_L_ and SI_S_, according to the following equation^11,17^:

1 $$ {\text{LSC }} = \, \left( {{\text{SI}}_{{\text{L}}} {-}{\text{ SI}}_{{\text{S}}} } \right) \, / \, \left( {{\text{SI}}_{{\text{L}}} + {\text{ SI}}_{{\text{S}}} } \right), $$

which is referred to as the Michelson contrast. In this study, the LSC was determined from five axial images acquired in the hepatobiliary phase, and the final measurement of the LSC was the average of five LSC values calculated from these five images.

The analyses of the HH15 and LHL15 were performed on Xeleris 3.0 Functional Imaging Workstation (General Electric Healthcare, Tokyo, Japan). The HH15 was calculated using cardiac counts at 3 and 15 min (H3 and H15, respectively) after the intravenous injection of ^99m^Tc-GSA, according to the following equation^5^:

2 $$ {\text{HH15 }} = {\text{ H15 }}/{\text{ H3}}{.} $$

The ROI used to measure the cardiac counts was set to surround both ventricles, in order to obtain the largest possible measurement area^5,20^. The LHL15 was calculated using the H15 and the liver counts at 15 min (L15) after the intravenous injection of ^99m^Tc-GSA, according to the following equation^5^:

3 $$ {\text{LHL15 }} = {\text{ L15 }}/ \, \left( {{\text{H15 }} + {\text{ L15}}} \right). $$

The ROI used to measure the liver counts was set to surround the entire liver^5^.

### ALBI score

The biochemical tests of serum bilirubin and albumin were assessed within 2 weeks before and after MRI scanning. The value of indirect bilirubin was computed to be the difference between total and direct bilirubin. The ALBI score is calculated using the total bilirubin [μmol/L] and albumin [g/L], according to the following equation:

4 $$ {\text{ALBI score }} = \, \left( {0.{66 } \cdot {\text{ log}}_{{{1}0}} {\text{total bilirubin}}} \right) \, {-} \, \left( {0.0{85 } \cdot {\text{ albumin}}} \right), $$

which is a new objective index that enables the quantitative evaluation of liver function^18^. The ALBI Grade is the grading system for determining the hepatic function in HCC patients, and its cut points classified by ALBI scores are as follows: − 2.60 or less (Grade 1), more than − 2.60 to − 1.39 or less (Grade 2), and more than − 1.39 (Grade 3)^18^. In this study, the group of patients who were at Grade 1 but had neither chronic liver disease nor hepatocellular carcinoma were classified as ‘Normal’ on the comparison with the LSC or LHL15.

### Imaging-based clinical stage classification

There is a clinical stage classification based on the severity of chronic liver diseases (currently known as liver damage classification for making decisions concerning the treatment of Japanese HCC patients)^21^. The criteria of LHL15 corresponding to this clinical stages exists as follows: 0.942 ± 0.017 (Normal), 0.909 ± 0.044 (Mild [Stage I]), 0.844 ± 0.066 (Moderate [Stage II]), and 0.706 ± 0.112 (Severe [Stage III])^5^. These criteria are used as one of the evaluation standards of ^99m^Tc-GSA liver scintigraphy and have been supported for many years^14^. In this study, the eLHL15 and LHL15 were divided into these four groups using the threshold levels of 0.936, 0.880, and 0.790, and were compared using contingency tables and images.

### Statistical analysis

The LSC was compared to the LHL15 and HH15 using Pearson's correlation coefficients. Additionally, tests of no correlation were performed. Equally, their indices were compared with the ALBI score and its related laboratory parameters. The comparison of the LSC or LHL15 among ALBI grades was performed using analysis of variance (ANOVA) and Tukey's multiple comparison test. The linear regression analysis wherein the LSC is a variable was performed, and the accuracy of the eLHL15 was evaluated by the R^2^ and standard error (SE). The contingency table analysis between the eLHL15 and LHL15 was performed using chi-square (χ^2^) test and Cramer's coefficient of association (Cramer's V), and the degree of agreement was tested on imaging-based clinical stage classification. In all statistical tests, a *P* value of < 0.05 (two-tailed) was considered as statistically significant. For interval estimation, the 95% confidence interval (CI) was calculated. All statistical analyses were carried out using statistical software (Microsoft^®^ Excel 2010; Microsoft, Redmond, WA).

## Results

The correlation coefficients of the LSC with the LHL15 and HH15 were 0.740 and –0.572, the *P*-values were both < 0.00001, and the CIs were [0.669, 0.798] and [–0.660, –0.468], respectively. The correlation between the LSC and LHL15 was stronger than that between the LSC and HH15. The correlation coefficients of their indices with ALBI scores and laboratory parameters are shown in Table 2. The correlation between the LSC and total bilirubin was obviously stronger than that between the LSC and albumin. The ALBI score and direct bilirubin were significantly, moderately, and negatively correlated with both of the LSC and LHL15 (*P* < 0.00001). The correlation between the LSC and indirect bilirubin was obviously stronger than that between the LHL15 and indirect bilirubin. The comparative table of the ALBI Grades with the LSC and LHL15 is shown in Table 3. Between Normal and Grade 1, although the LSC had a significant difference (*P* < 0.05), the LHL15 had no significant difference (*P* > 0.05), on Tukey's multiple comparison test.

**Table 2:** Correlation coefficients of the liver–spleen contrast (LSC), receptor index (LHL15), and blood clearance index (HH15) with the albumin-bilirubin (ALBI) scores and laboratory parameters. *P* statistical significance. The 95% confidence interval is presented as numbers in square brackets.

Item	ALBI score	Albumin	Total bilirubin	Direct bilirubin	Indirect bilirubin
LSC	−0.624	0.523	−0.606	−0.590	−0.431
	*P* < 0.00001	*P* < 0.00001	*P* < 0.00001	*P* < 0.00001	*P* = 0.00002
	[–0.703, –0.529]	[0.412, 0.619]	[–0.689, –0.508]	[–0.676, –0.488]	[–0.541, –0.307]
LHL15	−0.647	0.569	−0.553	−0.628	−0.306
	*P* < 0.00001	*P* < 0.00001	*P* < 0.00001	*P* < 0.00001	*P* = 0.03338
	[–0.723, –0.557]	[0.465, 0.657]	[–0.644, –0.446]	[–0.708, –0.533]	[–0.431, –0.171]
HH15	0.522	−0.471	0.405	0.467	0.228
	*P* < 0.00001	*P* < 0.00001	*P* = 0.00010	*P* < 0.00001	*P* = 0.83506
	[0.411, 0.618]	[–0.574, –0.353]	[0.279, 0.517]	[0.347, 0.572]	[0.087, 0.359]

**Table 3:** Comparative table of the albumin-bilirubin (ALBI) Grade with the liver–spleen contrast (LSC) and receptor index (LHL15).

	ALBI grade				*P* values in ANOVA
	Normal	Grade 1	Grade 2	Grade 3	
	n = 24	n = 53	n = 55	n = 4	
LSC	0.429 ± 0.042	0.371 ± 0.054	0.294 ± 0.112	0.069 ± 0.102	*P* < 0.00001
	(0.009)	(0.007)	(0.015)	(0.051)	
LHL15	0.923 ± 0.021	0.908 ± 0.029	0.861 ± 0.067	0.673 ± 0.089	*P* < 0.00001
	(0.004)	(0.004)	(0.009)	(0.044)	

The ‘*Normal’* is a group of patients who are at Grade 1 but have neither chronic liver disease nor hepatocellular carcinoma. All data are expressed as the mean ± standard deviation (standard error). In Tukey's multiple comparison test, all pairs have a statistically significant difference (*P* < 0.05), except the relationship between Normal and Grade 1 in the LHL15.

*n* number of subjects, *ANOVA* analysis of variance, *P* statistical significance.

**Figure 3:**
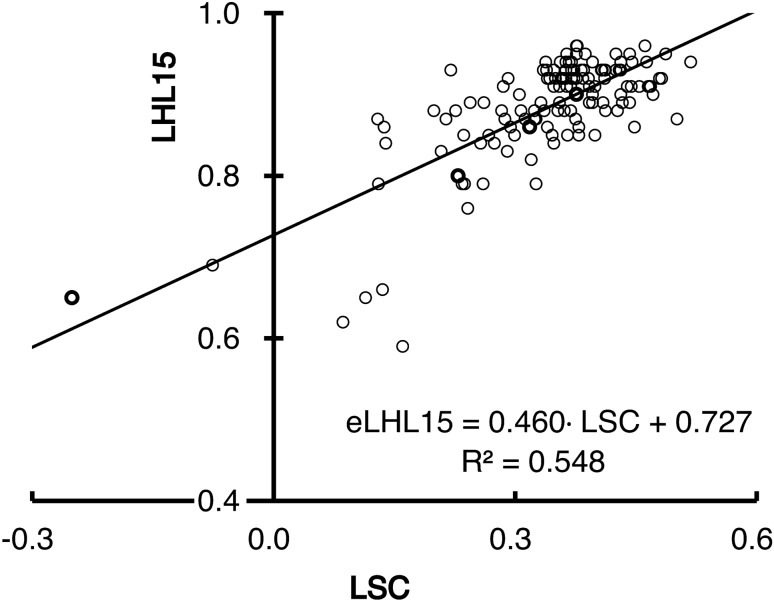
Relationship between the LSC and LHL15. The black line represents the regression line of the LSC and LHL15.

The regression equation of the LCS and LHL15 (Fig. 3) was as follows:

5 $$ {\text{eLHL15 }} = \, 0.{46}0 \, \cdot {\text{ LSC }} + \, 0.{727}{\text{.}} $$

For the slope and intercept, the *P*-values were both < 0.00001, and the CIs were [0.389, 0.532] and [0.702, 0.753], respectively. The test of no correlation was significantly rejected (*P* < 0.00001). The R^2^ was 0.548, and the SE of the eLHL15 was 0.045. Regarding imaging-based clinical stage classification, the contingency table between eLHL15 and LHL15 is shown in Table 4. There was significantly the correlation between eLHL15 and LHL15 (*P* < 0.00001), and the Cramer's V was 0.568. The degree of agreement on imaging-based clinical stages was 65.441%.

**Table 4:** Contingency table between the receptor index (LHL15) and the estimated LHL15 (eLHL15) from the liver–spleen contrast (LSC).

Item	Stage	LHL15			
		Normal	Mild	Moderate	Severe
eLHL15	Normal	5	6	1	0
	Mild	15	56	9	0
	Moderate	0	12	26	3
	Severe	0	0	0	4

The stages are based on imaging-based clinical stage classification. All data are expressed as the number of subjects. χ^2^(9) = 131.616, *P* < 0.00001, Cramer's V = 0.568, degree of agreement = 65.441%.

**Figure 4:**
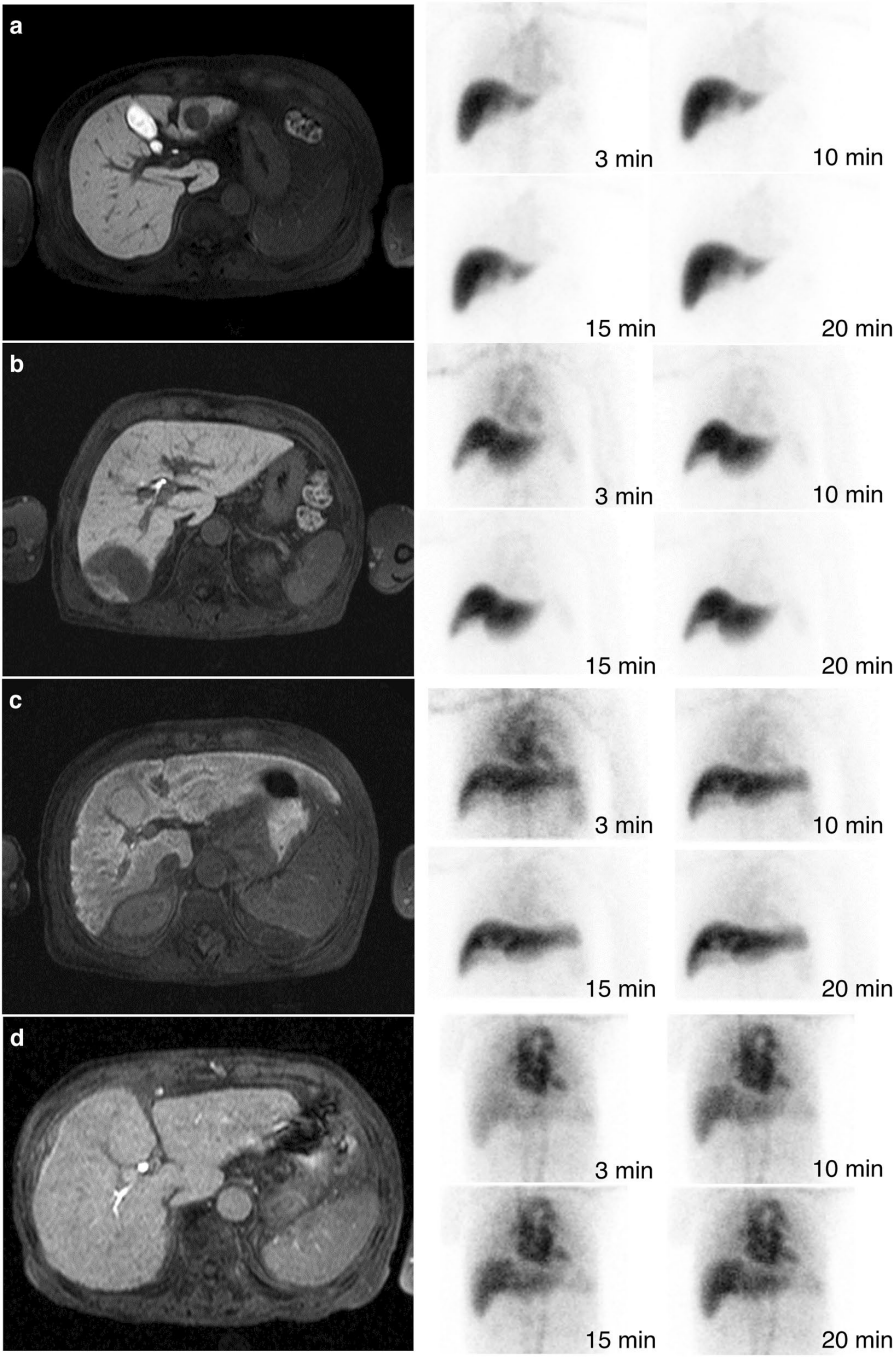
Reference images of various imaging-based clinical stages. The left and right image in figures are the hepatobiliary-phase images in Gd-EOB-DTPA-enhanced MRI and the planer images in ^99m^Tc-GSA liver scintigraphy, respectively. **(a)** Images of Normal stage. The LSC, eLHL15, LHL15, HH15, and ALBI score were 0.487, 0.95, 0.95, 0.61, and –2.899 (Grade 1), respectively. **(b)** Images of Mild stage. The LSC, eLHL15, LHL15, HH15, and ALBI score were 0.375, 0.90, 0.90, 0.61, and –2.756 (Grade 1), respectively. **(c)** Images of Moderate stage. The LSC, eLHL15, LHL15, HH15, and ALBI score were 0.257, 0.85, 0.84, 0.69, and –1.426 (Grade 2), respectively. **(d)** Images of Severe stage. The LSC, eLHL15, LHL15, HH15, and ALBI score were 0.114, 0.78, 0.65, 0.88, and –2.150 (Grade 2), respectively.

The LSCs on Normal, Mild, Moderate, and Severe stages were 0.477 ± 0.018 (SE = 0.005), 0.384 ± 0.033 (SE = 0.004), 0.263 ± 0.052 (SE = 0.008), −0.032 ± 0.168 (SE = 0.084), respectively. The reference images of various clinical stage are shown in Fig. 4. Regarding the Normal and Mild stages (Fig. 4a,b), the liver had nearly triple the SI (LSC ≒0.50) of the spleen and had over double the SI (LSC > 0.33) of the spleen in the hepatobiliary-phase images, respectively. In scintigraphy images, the nearly normal hepatic accumulation of ^99m^Tc-GSA was observed at 10 min after intravenous injection, and the faint pool of ^99m^Tc-GSA in the cardiac blood, which was observed until 10 min after intravenous injection, cleared at 15 min after intravenous injection. Regarding the Moderate stage (Fig. 4c), the liver did not have twice the SI (LSC < 0.33) of the spleen, but had a higher SI (LSC > 0) than the spleen in the hepatobiliary-phase image. In scintigraphy images, the liver and heart were visualized to the same SI at 3 min after intravenous injection, and the hepatic accumulation of ^99m^Tc-GSA which was delayed as compared with Normal and Mild stages had been finished at 20 min after intravenous injection. Regarding Severe stage (Fig. 4d), the liver had approximately the same SI (LSC ≒0) as spleen, or had a lower SI (LSC < 0) than the spleen in the hepatobiliary-phase image. In scintigraphy images, the liver uptake of ^99m^Tc-GSA was obviously delayed and had continued for 20 min after intravenous injection. In addition, the ^99m^Tc-GSA in cardiac blood remained even at 20 min after intravenous injection.

## Discussion

Gd-EOB-DTPA distributes non-specifically to the extracellular fluid (ECF), which is present in the intravascular space (blood plasma) and extracellular extravascular space (EES)^8,16,22^. Because the one-compartment model of Gd-EOB-DTPA in the ECF space of the liver (sinusoidal capillaries and Disse’s space) is very similar to that of the spleen (splenic blood and EES)^16^, the liver enhancement of the ECF approximates the spleen enhancement^22^. The peak enhancement of the spleen occurs approximately 1 min after intravenous injection^8,16,22^. After this peak, the SI_S_ decreases over 40 min, with washout of the contrast agent^8,16^. Therefore, we can assume that this washout is also occurring in the liver ECF space at 20 min after intravenous injection. A previous study that measured the LSC at 20 min after intravenous injection reported that the correlation between the LSC and HH15, which represents the retention of ^99m^Tc-GSA in the blood^5^, was stronger than that between the LSC and LHL15^12,13^. This fact may indicate that the LSC at 20 min after intravenous injection reflects enhancement by the Gd-EOB-DTPA remaining in liver ECF space rather than in the hepatocytes. In the present study, using the LSC measured at 60 min after intravenous injection, the correlation between the LSC and LHL15, which represents the hepatic accumulation of ^99m^Tc-GSA^5^, was stronger than that between the LSC and HH15^11^. These findings suggest that the LSC obtained at 60 min after intravenous injection more accurately reflects the uptake of Gd-EOB-DTPA into hepatocytes than the LSC obtained at 20 min after intravenous injection.

In addition, the 40% of the Gd-EOB-DTPA in the ECF is specifically taken up into the normal hepatocytes for biligenesis through the transporters on hepatocyte membranes: a Na^+^-taurocholate co-transporting polypeptide and the subfamilies of organic anion-transporting polypeptides. After uptake, Gd-EOB-DTPA actively excretes to biliary canaliculus by a multidrug resistance-associated protein (MRP) 2 that is used for bilirubin^22^. Through a decrease in the appearance of these transporters, non-conjugated bilirubin remains in the blood, and the uptake of Gd-EOB-DTPA is also suffocated. This phenomenon was supported by the strong correlation between the LSC and total (or indirect) bilirubin. In contrast, ^99m^Tc-GSA specifically binds to asialoglycoprotein receptors (ASGP-Rs) on the surface of hepatocyte membranes, and intracellular transport occurs through microtubules in the hepatocytes^5,12^. After uptake, ^99m^Tc-GSA and ASGP-Rs are hydrolyzed, and ASGP-Rs are recycled and returned to the membrane^23^. Since this recycling process has no concern with bilirubin transport, it appears that the LHL15 was not overly influenced by high total (or indirect) bilirubin^5^. However, both the LSC and LHL15 were strongly correlated with direct bilirubin. When the value of direct bilirubin rises, the reverse transport of conjugated bilirubin into the blood would actively occur via MRP 3^22^. This suggests the dysfunction of biliary canaliculus, which is caused by the cholestasis due to hepatocellular or cholangiocellular carcinoma^22^ or by the impaired expression of the hepatocyte in itself due to chronic hepatitis or liver cirrhosis^23^. Accordingly, both the LSC and LHL15 would fall in case of rise in direct bilirubin. The R^2^ of eLHL15 may be influenced by the difference in these two trafficking processes, but both the LSC and LHL15 sufficiently reflect liver function due to their strong correlation with ALBI scores.

Considering the difference in these trafficking processes, the evaluation with the LHL15 is recommended to patients with jaundice^5^. In contrast, only LSC had the ability to distinguish mild cases from normal ones regarding liver function, as well as the differential diagnosis of tumors. Thus, the evaluation of the eLHL15 should be applied to the follow-up examinations of patients who have chronic liver diseases. This is because it is possible to manage both tumor detection and liver function over time, such as biochemical tests, with tumor screening.

Regarding the accuracy of the eLHL15 predicted from the LSC, there is no problem because the R^2^ was > 0.5. Moreover, the correlation between the eLHL15 and LHL15 is strong because Cramer's V was > 0.5. Furthermore, the eLHL15 had a moderate agreement with the LHL15 on imaging-based clinical stage classification. These findings provide clear evidence that the eLHL15 has good reproducibility for the LHL15. Therefore, as the method clinically to evaluate the liver function with the eLHL15, we propose that this imaging-based classification is introduced into the gradation of the eLHL15 by using the criteria of the LHL15 as it is.

The events contaminating these evaluations was confirmed at the stage of collecting data in this study, and these images, a part of which is shown in Fig. 2b–d, was excluded from subjects. In MRI, there were motion artifacts due to respiration or peristalsis (Fig. 2b) and the image unevenness due to inhomogeneous fat suppression (Fig. 2c). Since these events cause the LSC to vary, measures such as reimaging are required. In scintigraphy, overvaluing of L15 was found to occur due to splenomegaly (Fig. 2d). This event is caused by containing the blood pool in the heart or spleen, which appears to overlap with the liver. However, as a rule, the ROI of L15 must cover the entire liver with a planar image^5^. Thus, care must be taken not to overestimate LHL15.

Our study using SIs has unavoidable limitation regarding quantitativity. The SIs, which are the sources of the LSC, depend on the used sequences and devices for MRI. In scintigraphy images, there is also the difference of the LHL15 between facilities^14^, which is caused by the differences in the collimator structure and the correction methods of scattered radiation between gamma cameras^24^. However, this difference itself on the LHL15 is not large enough to affect the diagnosis of liver function^14^. Future work should explore the correction method between different sequences or devices for MRI. If this work is completed, the diagnostic standard of the LSC to evaluate liver function would be established by a database constructed by multicenter studies. Until then, the method of this study, which is the conversion from the LSC to the eLHL15, seems to be very useful for clinicians.

## Conclusions

The LSC, not at 20 min, but at 60 min after injecting Gd-EOB-DTPA has a strong positive correlation with the LHL15, and this correlation coefficient was 0.740 (*P* < 0.00001) in this study. Although the LSC may just be influenced by high bilirubin values, the LSC correlates with the ALBI score to the same extent as the LHL15. The eLHL15 predicted from the LSC has sufficient accuracy clinically, and its R^2^ was 0.548 in this study. In addition, the eLHL15 has the good agreement with the LHL15 on the imaging-based clinical stage classification, which are applicable to the eLHL15. Furthermore, only LSC is able to separate the normal cases of liver function from Grade 1 of the ALBI Grades. Therefore, the eLHL15 can be used satisfactorily to evaluate liver function, instead of the LHL15.
